# Bone Morbidity in Childhood Leukemia: Epidemiology, Mechanisms, Diagnosis, and Treatment

**DOI:** 10.1007/s11914-014-0222-3

**Published:** 2014-07-02

**Authors:** Sogol Mostoufi-Moab, Jacqueline Halton

**Affiliations:** 1 Department of Pediatrics, The Children’s Hospital of Philadelphia, The University of Pennsylvania Perelman School of Medicine, 34th Street and Civic Center Boulevard, Philadelphia, PA 19104 USA; 2 Department of Pediatrics, The Children’s Hospital of Eastern Ontario, University of Ottawa, Ottawa, Ontario Canada K1H8L1

**Keywords:** Acute lymphoblastic leukemia, Hematopoietic stem cell transplant, Bone mineral density, Osteonecrosis, Vertebral fractures

## Abstract

Skeletal abnormalities are commonly seen in children and adolescents with leukemia. The spectrum ranges from mild pain to debilitating osteonecrosis (ON) and fractures. In this review, we summarize the skeletal manifestations, provide an update on therapeutic strategies for prevention and treatment, and discuss the most recent advances in musculoskeletal research. Early recognition of skeletal abnormalities and strategies to optimize bone health are essential to prevent long-term skeletal sequelae and diminished quality of life observed in children and adolescents with leukemia.

## Introduction

Skeletal development in childhood is characterized by sex, maturation, and race-specific increases in trabecular and cortical bone mineral density (BMD) and cortical dimensions [1]. This rapid accumulation of bone mass is dependent on the coordinated actions of growth factors and sex steroids in the setting of adequate biomechanical loading and nutrition. Thus, the growing skeleton is particularly vulnerable to the effects of childhood acute lymphoblastic leukemia (ALL) and hematopoietic stem cell transplant (HSCT) therapies and complications that suppress bone formation or promote bone resorption [2, 3].

Musculoskeletal abnormalities are well recognized in children with ALL at diagnosis, during treatment, and persist as long-term sequelae after treatment. The radiological abnormalities at diagnosis attributed to the leukemia process occur in up to 70 % of children [4–6]. Osteotoxic chemotherapy, steroid exposure, poor nutrition, low vitamin D, and poor muscle mass contribute to the development or worsening of bone pathology during therapy that may result in osteoporosis, fracture, and ON. In children after HSCT and/or cranial irradiation, endocrine abnormalities further contribute to the bone morbidity and altered quality of life.

Risk-directed therapy has substantially improved treatment outcomes for pediatric ALL, with 5-year survival rates approximating 80 % [7]. Given success of this magnitude, it is important to address the management and prevention of bone toxicity. Our knowledge of bone pathogenesis in childhood leukemia has advanced substantially over the past few decades and is the focus of this review.

## Skeletal Abnormalities at Leukemia Diagnosis

Radiographic abnormalities in childhood ALL have been described in the literature for over 80 years. Metaphyseal lucencies (ML) are the most common skeletal finding in most series, occurring in 7.5 %–70 % [4, 6, 8–10]. Although well recognized in children at the time of ALL diagnosis, ML are not pathognomonic for leukemia. They occur most frequently in the rapidly growing long bones, typically in the knees, ankles, and wrists [6].

Other radiographic skeletal lesions observed at the onset of leukemia include periosteal reaction (2 %–19 %) [6, 8, 9], osteolytic lesions (19 %–38 %) [6, 9], osteopenia (low bone mass on x-ray) (13 %–40 %) [8–10], and fractures (2 %–10 %) [8, 9]. Periosteal reaction occurs following leukemic infiltration between the periosteum and the cortex of the bone. As new bone is formed, there is elevation of the periosteum. Osteolytic lesions are seen as focal lucencies throughout the skeleton at the time of ALL diagnosis, occurring in the skull, pelvis, long bones, and small bones of the hands and feet [9]. Osteopenia, a more generalized condition, is reported at diagnosis and can be seen in children presenting with pathological fractures [8, 9].

The prevalence of non-vertebral fractures at ALL diagnosis is mostly limited by reported findings in retrospective studies and range from 3 % to 8 % [9, 11]. A single prospective study with radiographs limited to hips, knees, and ankles showed a prevalence of 10 % [8]. Similarly, vertebral fractures at the time of ALL diagnosis are more common than the previously reported fracture prevalence of 1.6 % based on a retrospective review of chest radiographs [12]. As noted by Halton et al the prevalence of severe vertebral compression fractures in children with newly diagnosed ALL is as high as 16 % [13]. Although back pain is a recognized complaint in children with vertebral fractures at ALL diagnosis, a significant proportion of these fractures (45 %) are asymptomatic, despite identified vertebral fractures on lateral spine imaging [13]. This is consistent with reported data in postmenopausal women with osteoporosis where vertebral compression fractures occur in the absence of clinical symptoms [14]. Overall, there is no identified relationship between vertebral fractures at ALL diagnosis with leukocyte count, leukemia risk category, or leukemia B or T subtype diagnosis [13, 15]. However, reduction in lumbar spine BMD Z-score at diagnosis is clearly associated with increased odds for vertebral fracture (OR 1.8; 95 % CI 1.10–2.9; *P* < 0.001) [13]. Bone fragility at the time of ALL diagnosis has been linked to increased skeletal resorption due to cytokine-mediated enhanced osteoclast activity [16••, 17].

## Bone and Mineral Homeostasis at Leukemia Diagnosis

Skeletal growth is unique to childhood and requires both modeling and remodeling. Bone modeling results in a net gain of bone volume, bone growth in length (through endochondral bone formation), and in width (the latter, through periosteal apposition) [18]. Remodeling occurs throughout life and maintains the integrity of bone and mineral homeostasis. Bone growth in length is not altered at leukemia diagnosis, as absolute height in children with leukemia is not different from healthy children [19]. However, bone density at leukemia diagnosis is altered prior to initiating chemotherapy. At diagnosis, serum markers of bone formation including osteocalcin, type I collagen carboxy terminal propeptide, and bone specific alkaline phosphatase are low [8, 20, 21]. Bone resorption markers, urinary N-telopeptide and type I collagen carboxy-terminal telopeptide, are normal or reduced in most studies but not all [8, 20, 21]. Reduced bone formation markers and low to normal resorption markers confirm a low bone turnover state. Histomorphometric assessment at diagnosis confirms the low bone turnover with reduced trabecular bone volume and reduced trabecular thickness [8, 22].

Defective mineralization of bone contributes to the bone pathology seen at leukemia diagnosis. Several studies have shown abnormally low levels of 1,25(OH)_2_D_3_, hypercalciuria, low parathyroid hormone, and low to normal levels of calcium, magnesium and phosphate [8, 20]. Histomorphometric biopsies are more sensitive than biochemical parameters for diagnosing mineralization defects [23]. The histormorphometric biopsies of the iliac crest at diagnosis show increased osteoid parameters suggestive of a delay in matrix mineralization (osteomalacia) in a subset of children [8]. Thus, the majority of children demonstrates altered bone mineralization and reduced bone formation with increased bone fragility prior to initiating osteotoxic therapy for the treatment of leukemia.

## Skeletal Abnormalities During Leukemia Therapy

ALL patients have increased incidence of fracture rates during treatment compared with healthy controls [24]. The reported incidence of nonpathologic fractures in ALL patients during treatment varies from 12 % [9] to 39 % [25]. Similarly, Van der Sluis et al noted a six-fold increase in fracture incidence in ALL patients during maintenance phase of treatment compared with healthy controls [26]. Furthermore, the incidence of nontraumatic vertebral fractures in children with ALL 1 year after initiation of chemotherapy is as high as 16 % (95 % CI 11 %–23 %), with 85 % of the incident vertebral fractures occurring in previously normal vertebral bodies [16••]. Importantly, more than 50 % of children with incident vertebral fractures demonstrated vertebral fractures at baseline, and the presence of at least one vertebral fracture at baseline was highly associated with increased odds of sustaining at least one incident vertebral fracture at 12 months (OR 7.30; 95 % CI 2.30–23.14; *P* = 0.001) [16••]. Children with identified incident vertebral fractures at 12 months of ALL treatment also demonstrate greater increases in spine BMD Z-scores between 6 and 12 months of ALL therapy, suggesting that the 12-month incident vertebral fractures may have occurred earlier than the reported observation period and subsequently followed by a degree of recovery manifesting as enhanced BMD accrual [16••].

During ALL treatment, bone formation and resorption markers are increased resulting in a decrease in total body BMD (mean -0.68, SD 1.26) within the first six months of treatment [25–27]. Longitudinal studies performed in children at the time of ALL diagnosis and during first year of treatment have revealed low lumbar spine BMD (-0.4 SD), [26, 28] with prevalence ranging from 12 %–17 % in various cross-sectional DXA-based studies [8, 26, 29]. Table 1 is a summary of studies examining BMD during ALL treatment [30]. Impaired BMD in childhood ALL is not only caused by the disease itself but also further impacted by the disease treatment. Corticosteroids, which play a critical role in ALL therapy, directly affect bone, and negatively impact the skeleton by altering the hormonal axis, intestinal calcium absorption, and renal excretion of calcium [31••]. While corticosteroids are the main culprit for skeletal toxicity in childhood ALL [25], other chemotherapeutic agents such as methotrexate and asparaginase additionally contribute to BMD deficits and reduced bone mineralization [31••, 32]. These agents impair osteoblast function, responsiveness and number, which subsequently account for the suppression of bone formation and contribute to the resultant osteopenia noted during and after ALL treatment [33].

**Table 1:** Summary of DXA studies of bone mineral density (BMD) during treatment for childhood acute lymphoblastic leukemia (ALL)

	Study design	N	Cranial XRT	Comments
Halton et al. [25]	Longitudinal 2 y tx	40	Y	↓ lumbar spine BMD from baseline in 47 % of children, ↓ lumbar spine BMC from baseline in 64 % of children. Compared w/ the status at dx, Z scores for BMD and BMC were not statistically significant throughout the 2 y of therapy.
Henderson et al. [28]	Longitudinal One y during tx or post-tx	37 ^a^	Y	↓ of ≥ 0.5 SD femoral BMD in 23 % of patients, ↓ lumbar spine BMD in 27 % of patients. Mean change in lumbar spine BMD z score –0.21 ± 0.14 and proximal femur –0.07 ± 0.12.
Arikoski et al.[27]	Longitudinal dx to 6 mo of tx	46 ^a^	Y	↓ (-2.1 %) lumbar spine BMAD from baseline, ↓ (–8.5 %) femoral BMAD from baseline, ↓ (-9.9 %) femoral BMD from baseline, ↓ (–0.7 SD) femoral BMD, ↓ (–0.7 SD) femoral BMAD, ↔ BMD lumbar spine.
Arikoski et al. [20]	Longitudinal dx to 1 y tx	28 ^a^	Y	↓ (–10.1 %) femoral BMAD from baseline, ↓ (–11.3 %) femoral BMD from baseline over first 12 mo after diagnosis.
Boot et al. [35]	Longitudinal dx, 6 mo, 1 and 2 y on tx, and 1 y post-tx	32	N	↓ (-1.1 SD) whole body BMD between dx and within first y of treatment, ↔ lumbar spine BMD during the first 6 mo after diagnosis.
van der Sluis et al. [26]	Longitudinal dx, during tx, and 1 y after tx	61	N	↓ (-0.68 SD) whole body BMD, ↓ (-0.65 SD) lumbar spine BMD, ↔ lumbar spine BMAD in the first 32 wk of treatment.
Davies et al. [36]	Longitudinal dx and 2 y tx	14	N	↓ femoral neck % BMC (82 %), ↓ femoral trochanter % BMC (72 %), ↓ lumbar spine % BMC (89 %), ↔ whole body % BMC etc.
Alos et al. [16••]	Longitudinal dx and 12 mo tx	155	N	∆ lumbar spine BMD Z-score from baseline to 6 mo - children w/o incident vertebral fractures at 12 mo: mean = 0.1, SD 0.8; children w/ incident vertebral fractures at 12 mo: mean = 0.1, SD = 0.9. ∆ lumbar spine BMD Z-score from 6 to 12 mo: w/o fractures: mean = 0.0, SD = 0.6, w/ fractures: mean = 0.3, SD = 0.5. ∆ lumbar spine BMD Z-score from baseline to 12 mo: w/o fractures: mean = 0.0, SD = 0.8; w/ fractures: mean = 0.3, SD = 1.1.

^a^Cohort included other childhood malignancies; ↑, increase; ↓, decrease; ↔, no change

*% BMC* % of control bone mineral content, *BMAD* bone mineral apparent density, *dx* ALL diagnosis, *mo* months, *tx* ALL treatment

Adapted from Davies et al [30]

Impaired BMD in ALL involves both the trabecular and cortical bone. Studies investigating bone geometry using peripheral quantitative computed tomography (QCT) in children during ALL treatment demonstrate a reduction in trabecular BMD in both weight bearing and nonweight bearing bones [34]. Previous DXA studies at the time of leukemia diagnosis have also demonstrated reduced lumbar spine BMD _(LS)_, which contains mostly trabecular bone, but with normal total body BMD _(TB)_ containing 80 % cortical bone [25–28, 35, 36]. While BMD_LS_ subsequently remains low during treatment, BMD_TB_ (cortical bone) declines rapidly during the initial phase of ALL therapy. Both BMD values show improvement shortly after cessation of ALL therapy but remain significantly lower compared with healthy children [26, 37••]. A positive linear relationship is noted with lower BMD_LS_ Z-scores at the time of ALL diagnosis and during therapy. Children with low BMD_LS_ at diagnosis continue to demonstrate low BMD_LS_ during the later phases of therapy [29]. Lower weight is a notable determinant of lower baseline BMD_LS_ at the time of ALL diagnosis [31••]. Older age at diagnosis is another independent risk factor for a more rapid decline of BMD_LS_ during ALL therapy, reflecting the effects of more intensified chemotherapy [26, 31••]. Moreover, reduced BMD has been demonstrated in many ALL survivors treated with chemotherapy alone and without cranial radiation exposure, highlighting treatment factors other than cranial radiation that negatively contribute to adverse BMD effects during treatment [37••, 38].

Lower BMD at diagnosis is associated with higher prevalence of subsequent bony fractures during ALL therapy [29]. More importantly, pediatric patients with ALL demonstrate a higher cumulative incidence of fracture rates during ALL therapy compared with healthy, age-matched children [9, 16••, 25, 26, 39, 40]. Low lumbar spine BMD at the time of ALL diagnosis (–1.3 SDS) and during treatment (–1.6 SDS), rather than the treatment-associated decline in BMD, is a strong determinant of markedly increased fracture risk during the first 3 years after ALL diagnosis, with 59 % of patients demonstrating clinically significant fractures (59 %) had lumbar spine BMD ≤ –2SD [31••]. Thus, low lumbar spine BMD at diagnosis and during treatment should be used to identify ALL patients at significant risk for bony fractures and osteoporosis [31••].

## Bone and Mineral Homeostasis During Leukemia Treatment

Serum markers of bone formation are suppressed with administration of glucocorticoids and multi-agent chemotherapy during ALL treatment [41]. Bone mineral accrual is adversely affected by low serum 1, 25 (OH)_2_D_3_ and hypercalciuria [25] Hypo- and hypermagnesemia associated with use of aminoglycosides and glucocorticoids during ALL treatment affect hydroxylation reactions necessary for production of 1, 25 (OH)_2_D_3_, and further contribute to mineralization defects present during ALL therapy [25, 42, 43].

## Osteopenia Prevention and Intervention

Physical activity combined with calcium and vitamin D supplementation in healthy children enhances bone mass accrual [44, 45]. Thus, promoting physical activity and exercise during and after ALL treatment, particularly given the marked reduced physical activity in some ALL survivors, may ameliorate bone mass acquisition during treatment. Additional nutritional supplementation with calcium and vitamin D may further augment the bone response to physical activity. However, Kaste SC et al reported no added benefit of cholecalciferol and calcium supplementation to nutritional counseling for improving lumbar spine BMD among adolescent and young adult survivors of ALL [46]. Anecdotal evidence exists for the use of bisphosphonates to improve BMD or treat bone fragility at diagnosis or during treatment of childhood ALL [47]. Given the small number of reported subjects and lack of randomized, controlled study design, the wider applicability of this approach deserves marked caution particularly as the effects of bisphosphonates on the leukemic disease process and treatment remain largely unknown. A key clinical question unanswered to date arising from the observation that reductions in BMD and prevalent vertebral fractures at diagnosis predict future fractures [16••, 48] is whether children with ALL and vertebral fractures early in the course of ALL treatment should be treated with bisphosphonates to prevent future fractures. To date there have been no randomized, placebo-controlled trials in pediatric ALL to provide adequate safety and efficacy data to justify this approach as standard of care. Thus, the use of bisphosphonates, at this time, remains limited to children with severe, symptomatic bone morbidity administered only on compassionate grounds [16••].

## Skeletal Recovery After Leukemia Therapy

To date, fractures among long-term childhood cancer survivors remain largely uncharacterized. While prior studies report fractures during ALL therapy or within the first few years of treatment cessation, it is unclear whether the alterations to bone metabolism during ALL therapy also impact post-therapy risk of fractures. Furthermore, most of these studies are limited by small sample size and, thus, unable to consider additional factors such as survivor demographics and life style on future fracture risk.

Nysom et al reported a fracture prevalence of 55 % in ALL survivors, median of 7.6 years (range 1.6–18.3 years) from completion of therapy. In this small, cross-sectional study, fractures were more common in younger participants (<20 years old) compared with controls, with no fractures noted among survivors within the oldest age group (>29 years). Furthermore, none were spinal compression fractures [49]. In a subsequent retrospective study, the 5-year cumulative incidence of fractures in ALL survivors was 28 ± 3 %, with 85 % of fractures occurring during maintenance phase or shortly after ALL treatment cessation. The median time to fracture was 15 months from diagnosis (interquartile range 10–24 months) [50], and majority of fractures occurred in long bones with only 4 reported vertebral fractures. The cumulative incidence was higher in males (37 ± 5 %) and patients ≥9 years of age (46 ± 8 %), suggesting increased fracture rates in adolescent males after ALL treatment, possibly from participation in organized sports or risk-taking behaviors [50]. On the other hand, in the largest cross-sectional study to date, with a median of 22.7 years of follow-up from cancer diagnosis, the childhood cancer survivor study reported a comparable fracture prevalence ratio between female ALL survivors and their siblings (0.99; CI 95 % 0.88–1.12; *P* = 0.87), while among males, the prevalence ratio of fractures was lower among survivors relative to the sibling control group (0.91; 95 % CI 0.83–1.00; *P* = 0.05) [51••]. A recognized limitation of this study is use of self-report questionnaires to collect information on fracture occurrence and health-related information among survivors of childhood cancer and their siblings. In addition, interpreting these results requires caution as majority of ALL survivors have yet to reach an age where the underlying population risk of fracture increases substantially. Likewise, the frequency of permanent vertebral deformity arising from prior vertebral fractures and its long-term sequelae in ALL survivors remain unknown. Therefore, future longitudinal studies in long-term ALL survivors are critical to better delineate the effects of chemotherapy and radiation on bone health of aging survivors.

After completion of ALL treatment with chemotherapy alone, no significant long-term decreases in BMD are present in longitudinal follow-up studies [37••, 52]. Importantly, low trabecular BMD improves significantly after completion of ALL therapy. On the other hand, early increases in cortical dimensions are associated with a decline in cortical BMD; however, over time, ALL survivors demonstrate stable cortical dimensions with an increase in cortical BMD, reflecting the time necessary to mineralize newly formed bone [37••]. On the other hand, patients treated with cranial or spine radiation continue to demonstrate significantly reduced total body and lumbar spine BMD long after completion of ALL therapy [53–55], with high dosage (≥24 Gy) of cranial or craniospinal radiation the strongest identified risk factor for persistently low BMD (Z-score ≤ -1) during young adulthood [56]. Defects in the hypothalamic-pituitary axis leading to abnormalities in growth hormone production, direct radiation effects on developing bone, as well as inadequate gonadal function are probable causes leading to this prolonged disturbance of bone metabolism. Majority of BMD differences in adult ALL survivors compared with healthy controls stem from reduced adult height in ALL survivors after cranial radiation, with the DXA-derived values no longer significantly abnormal once they are adjusted for the short stature [55]. However, spine QCT measures of trabecular BMD in ALL survivors (median 31 years old) treated with cranial or craniospinal radiation demonstrate a 5.7 % prevalence of BMD Z-score ≤ –2, suggesting long-standing effects of suboptimal BMD in this select group of patients [56]. Additional potential identified risk factors contributing to low BMD in adult ALL survivors include inadequately treated hypogonadism, vitamin D deficiency, low physical activity, and inadequate growth hormone production. These risk factors can provide guidance in identifying and targeting specific adult ALL survivors at marked risk for skeletal complications during adulthood. However, the majority of younger children treated with chemotherapy alone recover from BMD deficits over time with no lasting deficits into young adulthood [52, 56]. Given that ALL is the most common malignancy of childhood, these findings are reassuring and highlight that the most significant skeletal impact of ALL is at the time of diagnosis followed by active phase of treatment with substantial recovery following completion of therapy.

## Skeletal Abnormalities after Hematopoietic Stem Cell Transplantation

The pathogenesis of bone deficits in pediatric HSCT recipients is multifactorial. HSCT myeloablative regimens can directly damage the recipient’s osteoprogenitor cells within the bone marrow, negatively affecting bone formation [57]. Survivors of allogeneic HSCT demonstrate additional risk factors for poor bone health, including malnutrition [42], reduced muscle strength [58], chemotherapy [41], total body irradiation, immune suppressive therapies, and sex hormone deficiencies [59]. Graft Vs Host Disease (GVHD) and dysregulation of the immune system serve as additional threats to bone health, due to osteoclast activation, and reduced osteoblast number as well as function [60]. These risk factors have lasting effects that persist long after HSCT treatment [61•]. The true fracture incidence after childhood HSCT is not known, and only a few cross-sectional studies to date have actually examined the prevalence of fracture rates in long-term pediatric HSCT recipients [61•, 62, 63]. While an increased prevalence of fracture rates is not identified in long-term survivors of childhood HSCT, these few studies are limited by cross-sectional design, small sample size, and the use of self-report for identifying fractures. Therefore, the true incidence of clinically asymptomatic fractures remains poorly delineated in this increasing population at significant risk for inadequate bone accrual and metabolism.

DXA-based studies in children and adults within the first year following allogeneic HSCT have demonstrated substantial bone deficits, in conjunction with elevated markers of bone resorption, and low markers of bone formation [64–66]. However, there are only a few studies specifically assessing BMD in survivors of pediatric allogeneic HSCT, all with variable findings (Table 2) [64, 67–70]. In one of the earliest studies, Bhatia et al reported DXA total body BMD deficits (median Z-score -0.5) in a small sample of 10 pediatric patients, ages 3–18 years, and a median of 2 years following allogeneic HSCT. Unfortunately, this study was limited by lack of data on the effect of growth failure on DXA BMD Z-scores relative to age [71]. In contrast, Nysom and colleagues reported no difference in height-adjusted DXA whole-body BMD and bone mineral content in 25 survivors of allogeneic HSCT 4–13 years after HSCT compared with healthy controls [68]. However, as noted previously, DXA methods adjusting for BMD Z-scores without consideration of age results in an underestimation of bone deficits, which likely accounts for the absence of bone deficits reported by Nysom and colleagues in this study [68, 71].

**Table 2:** Summary of DXA studies of low bone mineral density (BMD) after childhood allogeneic HSCT

	N	Study design	Age at HSCT (y)	Age at study (y)	Follow-up^a^ (y)	DXA Z-score		*P* value ^b^	Height Z-score
						Total body BMD	Lumbar spine BMD		
Bhatia et al. [64]	10	Cross-sectional	5 (3–18) ^c^	12 (4–22) ^c^	2 (1–10)	–0.5 (–2.0 to 1.0) ^c^		0.05	
Nysom et al. [68]	25	Cross-sectional	11 (6–18) ^c^	18 (11–27) ^c^	7 (4–13)	–0.5^c^	–0.5 ^c^	0.08; 0.54	
Petryk et al. [69]	21	Longitudinal	10 (5–18) ^d^	10 (5–18) ^d^	≤ 1		^**ƒ**^–0.9 (–2.9 to 1.1) ^d^	0.022	
Perkins et al. [70]	17	Cross-sectional	13 (4–24) ^d^	13 (4–24) ^d^	12 (3–22) ^d^		–0.3 (–2.4 to 2.0) ^d^		–1.8

*HSCT* hematopoietic stem cell transplant

^a^Follow-up y since HSCT

^b^ *P* value compared with healthy controls

^c^Median (range)

^d^Mean (range)

^**ƒ**^Represents value at 1 year follow-up time point since HSCT compared with baseline; authors stated no change in lumbar spine BMD after adjusting for 1 year change in height SD but actual results not reported.

As DXA is a two-dimensional technique that combines trabecular and cortical bone mass within a projected bone area, it does not allow for discrete measures of trabecular and cortical volumetric BMD or cortical dimensions. On the other hand, QCT is a three-dimensional technique, which distinguishes between cortical and trabecular bone. To date, only two studies utilize QCT to examine trabecular BMD and cortical dimensions following HSCT [61•, 67]. In a study by Kaste and colleagues, significant deficits were reported in QCT measures of spine trabecular volumetric BMD in 48 allogeneic HSCT survivors (median Z-score -0.88) a median of 5 years after HSCT. These deficits were not associated with gender, age at HSCT, interval since HSCT, conditioning regimen, or endocrine dysfunction in survivors [67]. Subsequently, Mostoufi-Moab et al reported substantial growth failure (median Z-score -1.21), low trabecular volumetric BMD (median Z-score -1.05), smaller cortical dimensions (median section modulus Z-score -0.63 even after adjustment for shorter tibia length), and marked cachexia (median muscle cross-sectional area Z-score –1.01) in 50 long-term survivors of allogeneic HSCT (range 3–16 years since HSCT) using peripheral QCT compared with a large healthy reference population [61•]. The magnitude of these deficits exceeded those observed in children with active Crohn’s disease [72], juvenile rheumatoid arthritis [73], and chronic kidney disease [74], highlighting the lasting impact of allogeneic HSCT and its therapies. Importantly, the vast majority of the HSCT recipients in this study had not been treated with corticosteroids or immune suppressive medications for many years. Furthermore, the study delineated discrete associations between TBI, growth hormone deficiency (GHD), and trabecular and cortical deficits despite appropriate hormone replacement in subjects with a diagnosis of endocrinopathy (median trabecular BMD Z-score after TBI -1.30 vs -0.49 without TBI). Allogeneic HSCT survivors also demonstrated significant pubertal delay as expected from treatment-related toxic gonadal effects. Thus, treatment-associated GHD and hypogonadism further contribute to compromised skeletal acquisition in pediatric allogeneic HSCT survivors. While both studies were limited by cross-sectional design, they demonstrate striking bone deficits years after HSCT and completion of therapy. Future longitudinal studies are necessary to determine if these deficits progress or recover over time and to identify associations with fracture.

## Osteonecrosis

ON is a serious and debilitating complication of ALL and its treatment. The etiology of developing ON in ALL is complex and attributed, at least in part, to the use of high dose corticosteroids [75]. A myriad of pathophysiologic mechanisms have been postulated including altered bone and lipid metabolism and thrombophilia. The final common pathway in the development of ON is compromised blood flow resulting in infarction and necrosis of the bone [76]. ON results from: (1) direct suppression of osteoblasts and apoptosis of osteocytes, (2) stimulation of intramedullary lipocyte proliferation and hypertrophy within the bone marrow, resulting in reduced blood flow, and (3) further stasis and ischemia due to impact on vascular endothelial and smooth muscle cells [77–79].

Over the past 2 decades, ON has become the focus of numerous studies to determine the extent, onset, cause, and outcome of this disorder. The prevalence has been reported in several retrospective and prospective series from cooperative leukemia clinical trial groups as well as large single institutions (Table 3). Prevalence and incidence rates vary widely due to the study population [high risk patients (HR) vs combined HR and standard risk (SR) patients], retrospective vs prospective, symptomatic vs asymptomatic, and by method of detection. Cumulative incidence of symptomatic ON at the end of leukemia therapy occurs in 0.9[80] to 17.6 % [81••] while asymptomatic ON occurs in up to 53.9 % [81••]. In symptomatic children, the clinical course is often multi-articular and bilateral with the hips and knees most commonly affected [75, 82]. Recent studies using whole body MRI assessing the entire skeleton have shown widespread multifocal lesions affecting upper and lower extremity joints (hips, knees, shoulders), long bones, and 10 % of lesions involved the small bones of the hands and feet [83]. The natural history of development and progression of ON in pediatric leukemia are poorly understood. Predictors associated with joint collapse and need for arthroplasty include older age at diagnosis, pain, area of ON >30 % and lesions close to the articular surface [84, 85]. The clinical significance of asymptomatic ON in children is unclear as the lesions may resolve, remain stable or progress [86, 87••].

**Table 3:** Summary of studies of osteonecrosis in childhood acute lymphoblastic leukemia

Reference	Study population	#ON/#pts risk category	Percentage of patients with ON			Diagnosis of ON
			Total^c^	<10 y	>10 y	
	Retrospective					
Mattano et al. [82]	CCG-1882	111 of 1409	9.3 %	0.9 %	14.2 %	Y 1–32 %
	Symptomatic	HR				Y 2–54 %
						Y 3–13 %
Strauss et al. [50]	DFCI 87-01, 91-01	13 of 176	7 %	4 %	21 %	Median- 14 mo
	Symptomatic	SR + HR				Continuation 45 %
						S/P Treatment 33 %
Arico et al. [89]	AIEOP-ALL 95	15 of 1421	1.6 %		7.4 %	Median 17 mo
	Symptomatic	SR + IR + HR				
Bűrger et al. [75]	ALL-BFM 95	31 of 1951	1.8 %	0.2 %	8.9 %	Y 1–35 %
	Symptomatic	SR + MR + HR				Y 2–32 %
						Y 3–29 %
Elmantaser et al. [90]	UKALL97, 01, 03	18 of 186	9.7 %	N/A	N/A	17 mo SAC
	Symptomatic	SR + HR + TBI				46 mo TBI
Hyakuna et al. [94]	JCCLSG	16 of 1095	0.76 %(941)	0.42 %	15.6 %	N/A
	ALL 941. 2000, 2004	SR + HR	0.35 %(2000)			
	Symptomatic		3.6 %(2004)			
	Prospective					
Ojala et al. [86]	Nordic Protocol	9 of 24	38 %	<5 y 0.08 %	>5 y 38 %	Median 12 mo
	Asymptomatic	HR + IR + SR				Range 8–25 mo
	Single Institution					
Ribeiro et al. [93]	St Jude XIIIA	17 of 116	15.5 %	N/A	N/A	N/A
	Asymptomatic-Symptomatic	NHL + HR				Most S/P therapy
Mitchell et al. [80]	MRC ALL97, 99	15 of 1603	0.9 %	N/A	N/A	Second phase of therapy
	Symptomatic	SR + HR				
Kawedia et al. [81••]	St Jude total XV	259 of 364	71.8%^b^,^a^	10 % ^a^	44.6 % ^a^	Y 1– all symptomatic patients
	Asymptomatic^b^		53.9%^b^			
	Symptomatic^a^		17.6 % ^a^			
Mattano et al. [98]	COG AALL0232	110 of 1647	10.4 %	2.6 %	15.2 %	N/A
	Symptomatic	HR				
Vora et al. [96]	UKALL2003	NA of 1864	4 %	1 %	10-15 y 13 %	Maintenance
	Symptomatic	SR + HR			>16 y 16 %	
Mőricke et al. [97]	ALL-BFM 2000	111 of 3048	3.6 %	♀ 0.8 %	♀18.4 %	15 mo
	Symptomatic			♂0.7 %	♂ 7.6 %	Range 2–73 mo
Vrooman et al. [95]	DFC1 00-01	23 of 492	6 %	3.5 %	14 %	N/A
	N/A	SR + HR				
te Winkel et al. [87••]	DCOGALL9	38 of 694	6.1 %	N/A	N/A	Mean 1.2 y
	Symptomatic	NHR + HR				Range 0.1–2.7 y
Mattano et al. [91]	CCG 1961	143 of 2056	7.7 %	1 %	11.9 %	<2 y-88 %
	Symptomatic	HR				S/P therapy–10 %

Adapted from te Winkel et al. [87••]

*AIEOP* Associazione Italiana Di Ematologia, *BFM* Berlin Frankfurt Munster, *CCG* Children’s Cancer Group, *DCOG* Dutch Child Oncology Group, *DFCI* Dana Farber Cancer Institute, *ED* Oncologia Pediatrica, *HR* high risk, *IR* intermediate risk, *JCCLSG* Japanese Childhood Cancer and Leukemia Study Group, *MR* medium risk, *MRC* Medical Research Council, *NA* not available, *NHR* nonhigh risk, *SAC* standard active chemotherapy, *S/P* status post, *SR* standard risk, *TBI* total body irradiation, *UKALL* United Kingdom ALL; *#ON* number of patients with osteonecrosis; *#pts* number of patients enrolled on the leukemia clinical trial

^a^Symptomatic

^b^Asymptomatic

^c^ *<10y%* plus >10y% does not equal total as there are more children in the <10 age group

The onset of ON is highly variable with several case reports of ON detected at diagnosis [88]. In most series, the majority of children present within the first 2 years from diagnosis [75, 81••, 82, 89–91]. Magnetic resonance imaging (MRI) is a sensitive tool for detecting ON at earlier stages and in asymptomatic locations [81••]. Research from St. Jude’s Hospital using a prospective MRI surveillance study of ON identified all symptomatic patients within the first year of ALL diagnosis. The cumulative incidence at one year was 14.6 % for grade 2–4 ON and 35.4 % for grade 1 ON. The presence of ON at initial MRI screening performed during the first 6–8 months of ALL therapy was the most robust predictor of subsequent ON progression [81••]. Patients with grade 1 ON at first MRI screening were more likely to develop symptomatic grade 2–4 ON (26 %) compared with patients initially negative for ON (14 %) [81••].

In most series, age >10 years [50, 75, 81••, 82, 89, 91–98], female sex [80, 82, 87••, 89, 97], and Caucasian race [82, 92] are identified risk factors for developing ON. Increased BMI [99] is another recognized risk factor in some but not all studies [93]. Cumulative exposure to steroids is a key factor in the development of ON, and corticosteroids are the essential component of childhood ALL therapy. Improvements in event free survival have been attributed to the more potent antileukemic effect of dexamethasone compared with prednisone [80, 95]. However, the frequency of ON is greater with ALL treatment regimens using dexamethasone [82, 90, 95]. Burger et al reported on the prednisone equivalent dose from three leukemia cooperative group trials and showed that the higher incidence of ON was associated with a higher total steroid dose [75]. Mattano reported that ALL patients in the HR group receiving two dexamethasone pulses exhibited a 1.4-fold higher increase in symptomatic ON than those who received a single dexamethasone pulse [82]. Dexamethasone dosing schedule may also be a factor in the development of ON. Alternate-week dexamethasone dosing has shown a lower incidence of ON (11.8 % vs 23.2 %) compared with continuous dexamethasone dosing [91].

Recent studies have reported predictive biomarkers identifying patients at genetic risk of developing ON. Single nucleotide polymorphism (SNPs) arrays have identified multiple candidate genes including PAI-1(SERPINE 1) [100], VDR [92], and CYP3A4 [101], with conflicting results [100, 102]. More recently a locus on chromosome 2 encoding for ACP1 and SH3YL1(which regulates lipid levels and osteoblast differentiation) have been identified as a biomarker for symptomatic ON risk. Furthermore, the same SNPs have an association with a phenotype of higher cholesterol and lower albumin, independent risk factors for ON [81••]. However, thrombophilia testing including Factor V Leiden, Prothrombin 20210G→A and methylene tetrahydrofolatereductase (MTHFR,677C—T) were not associated with ON [103].

Similarly, pediatric HSCT recipients are at high risk for the development of ON with prevalence as high as 44 % in HSCT survivors screened by MRI [67, 104]. However, the true prevalence of ON is unknown, as the majority of HSCT recipients do not undergo prospective screening assessment with MRI, which is a more sensitive method for detecting ON [105]. The reported median interval for development of ON in pediatric HSCT recipients is 11 months after HSCT[106] with the earliest time point from 1 to 6 months after the onset of glucocorticoid therapy for the treatment of GVHD [107, 108]. Patients typically present with vague, diffuse bone pain with majority manifesting ON in 2 or more joints [107]. Once again, pathogenesis is multifactorial and corticosteroids are the strongest risk factor for ON with both the cumulative dose as well as the duration of treatment playing a role [76, 109]. Higher incidence of ON is present in TBI-based conditioning regimens likely due to radiation-induced micro-vascular damage [77]. The risk of ON increases with GVHD, possibly from GVHD-related increased risks for microangiopathy [78]. Notable overlap exists between risk factors for low BMD and ON following pediatric HSCT, with both conditions coexisting in pediatric HSCT survivors as they share similar pathogenic pathways and likely have additive effects [85]. Thus, the impaired osteoblast activity after HSCT can contribute to reduced BMD and the reduced number of osteoblasts can in turn adversely affect the regenerative potential of the osteogenic compartment over the course of ON after pediatric HSCT [108]. Future, prospective studies are necessary to not only identify the exact incidence and timing of ON occurrence after HSCT, but also discern effective and safe pharmacologic interventions in ON prevention as well as treatment in pediatric HSCT survivors.

## ON Prevention and Treatment

Despite improved survival of childhood ALL, treatment-related skeletal complications such as ON significantly contribute to short- and long-term disability in many survivors of pediatric hematologic malignancies and HSCT [84]. Given the variable natural history of ON, treatment decisions have been equally difficult with notable lack of established standardized regimens. Current treatment options for ON include analgesia, [84] limited weight bearing, physical therapy, and surgical procedures including core decompression [110] as well as joint replacement [111]. Other newer surgical techniques include the use of vascularized bone grafts [112], and the combination of core decompression with the insertion of human bone morphogenetic protein [113]. While surgery still remains the conventional approach, there is concern about the use of surgical interventions in growing pediatric patients with open physes [82, 114].

Additional alternative treatments have included external stimulation/capacitance coupling [115, 116] as well as the use of hyperbaric oxygen, all without any established clear benefits [117]. A number of medical therapies have also been undertaken with variable and inconsistent results including the use of calcium channel blockers (nifedipine), prostaglandin infusions, low molecular weight heparin, and statins [118–121]. These pharmacologic agents predominantly ameliorate the regulation of blood supply targeting local ischemia [122] or lipocyte proliferation [120]. More recently, several studies with small number of adult and pediatric subjects have reported the use of bisphosphonates for the treatment of ON [123–126]. The rationale for the use of bisphosphonates stems from the prevention of osteoclast bone resorption during the revascularization and uncoupled bone remodeling phase in the ON bone, and, thus, preserving bone shape; [84, 127] however, the data on the clinical and radiological outcome of children with chemotherapy-associated ON treated with bisphosphonates remains limited [84, 128•, 129].

While treatment with bisphosphonates contributes to pain improvement with a reduced requirement in oral analgesia, their use has failed to demonstrate the prevention, destruction, and subsequent collapse in hip joints, which is the most affected of weight-bearing joints [84]. This limited effectiveness of bisphosphonates in treatment of hip ON might reflect inadequate drug distribution to areas of necrotic bone [84] However, limited data in one study using zoledronic acid in a small group of patients suggested slowed progression of joint destruction predominantly in knee joints [84]. Unfortunately, children and adolescents with advanced ON joint involvement at the end of cancer treatment are more likely to develop early osteoarthritis and require joint replacement early in adult life, with the relative risk of major joint replacement in long-term survivors of childhood cancer compared with siblings reported at 54 % (95 % CI 7.6–38.6) [130]. Thus, given that treatment with bisphosphonates in cancer survivors remains safe without any significant side effects, future novel treatment approaches and strategies, such as prophylactic administration of bisphosphonates, might prove more effective in reduction of the frequency and severity of ON during treatment for childhood hematologic malignancies or HSCT [131].

## Conclusions

In summary, the growing skeleton is vulnerable to the leukemic process and its osteotoxic therapy. Early recognition and intervention strategies to optimize bone health are essential to prevent long-term sequelae from osteopenia, fractures and osteonecrosis observed in children and adolescents with leukemia. DXA assessments of BMD at the time of leukemia diagnosis and the presence of vertebral compression fractures should be used to predict bone health during treatment. Following cessation of leukemia treatment or HSCT, survivors require continued surveillance for skeletal morbidities into late adulthood with specific attention to bone health including optimization of nutrition, mobility, and exercise. Future studies are necessary to examine the impact of interventions such as bisphosphonate therapy, exercise, and nutritional supplements both during and following leukemia treatment.

## References

[1] Leonard MB, Elmi A, Mostoufi-Moab S (2010). Effects of sex, race, and puberty on cortical bone and the functional muscle bone unit in children, adolescents, and young adults. J Clin Endocrinol Metab.

[2] Leonard MB (2007). Glucocorticoid-induced osteoporosis in children: impact of the underlying disease. Pediatrics.

[3] Sun L, Blair HC, Peng Y (2005). Calcineurin regulates bone formation by the osteoblast. Proc Natl Acad Sci U S A.

[4] Baty JM, Vogt EC (1935). Bone changes of leukemia in children. Am J Roentgenol.

[5] Simmons CR, Harle TS, Singleton EB (1968). The osseous manifestations of leukemia in children. Radiol Clin N Am.

[6] Willson JK (1959). The bone lesions of childhood leukemia; a survey of 140 cases. Radiology.

[7] Pui CH, Robison LL, Look AT (2008). Acute lymphoblastic leukaemia. Lancet.

[8] Halton JM, Atkinson SA, Fraher L (1995). Mineral homeostasis and bone mass at diagnosis in children with acute lymphoblastic leukemia. J Pediatr.

[9] Rogalsky RJ, Black GB, Reed MH (1986). Orthopaedic manifestations of leukemia in children. J Bone Joint Surg Am.

[10] Silverman FN (1948). The skeletal lesions in leukemia; clinical and roentgenographic observations in 103 infants and children, with a review of the literature. Am J Roentgenol Radium Ther.

[11] Riccio I, Marcarelli M, Del Regno N (2013). Musculoskeletal problems in pediatric acute leukemia. J Pediatr Orthop B.

[12] Ribeiro RC, Pui CH, Schell MJ (1988). Vertebral compression fracture as a presenting feature of acute lymphoblastic leukemia in children. Cancer.

[13] Halton J, Gaboury I, Grant R (2009). Advanced vertebral fracture among newly diagnosed children with acute lymphoblastic leukemia: results of the Canadian Steroid-Associated Osteoporosis in the Pediatric Population (STOPP) research program. J Bone Miner Res.

[14] Dennison E, Cooper C (2000). Epidemiology of osteoporotic fractures. Horm Res.

[15] Clausen N, Gotze H, Pedersen A (1983). Skeletal scintigraphy and radiography at onset of acute lymphocytic leukemia in children. Med Pediatr Oncol.

[16.••] Alos N, Grant RM, Ramsay T (2012). High incidence of vertebral fractures in children with acute lymphoblastic leukemia 12 months after the initiation of therapy. J Clin Oncol.

[17] Jayanthan A, Miettunen PM, Incoronato A (2010). Childhood acute lymphoblastic leukemia (ALL) presenting with severe osteolysis: a model to study leukemia- bone interactions and potential targeted therapeutics. Pediatr Hematol Oncol.

[18] Jaworski ZFG, Kimmel DB, and Jee WSS. Cell kinetics underlying skeletal growth and bone turnover. In: Recker RR, editor. Bone histomorphometry: techniques and interpretation. Boca Raton: CRC Press; 1983. p. 226–7.

[19] Robison LL, Nesbit ME, Sather HN (1985). Height of children successfully treated for acute lymphoblastic leukemia: a report from the Late Effects Study Committee of Childrens Cancer Study Group. Med Pediatr Oncol.

[20] Arikoski P, Komulainen J, Riikonen P (1999). Alterations in bone turnover and impaired development of bone mineral density in newly diagnosed children with cancer: a 1-year prospective study. J Clin Endocrinol Metab.

[21] Sorva R, Kivivuori SM, Turpeinen M (1997). Very low rate of type I collagen synthesis and degradation in newly diagnosed children with acute lymphoblastic leukemia. Bone.

[22] Leeuw JA, Koudstaal J, Wiersema-Buist J (2003). Bone histomorphometry in children with newly diagnosed acute lymphoblastic leukemia. Pediatr Res.

[23] Arnala I (1991). Use of histological methods in studies of osteoporosis. Calcif Tissue Int.

[24] Hogler W, Wehl G, van Staa T (2007). Incidence of skeletal complications during treatment of childhood acute lymphoblastic leukemia: comparison of fracture risk with the General Practice Research Database. Pediatr Blood Cancer.

[25] Halton JM, Atkinson SA, Fraher L (1996). Altered mineral metabolism and bone mass in children during treatment for acute lymphoblastic leukemia. J Bone Miner Res.

[26] van der Sluis IM, van den Heuvel-Eibrink MM, Hahlen K (2002). Altered bone mineral density and body composition, and increased fracture risk in childhood acute lymphoblastic leukemia. J Pediatr.

[27] Arikoski P, Komulainen J, Riikonen P (1999). Impaired development of bone mineral density during chemotherapy: a prospective analysis of 46 children newly diagnosed with cancer. J Bone Miner Res.

[28] Henderson RC, Madsen CD, Davis C, Gold SH (1998). Longitudinal evaluation of bone mineral density in children receiving chemotherapy. J Pediatr Hematol Oncol.

[29] Rayar MS, Nayiager T, Webber CE (2012). Predictors of bony morbidity in children with acute lymphoblastic leukemia. Pediatr Blood Cancer.

[30] Davies JH, Evans BAJ, Jenney MEM, Gregory JW (2005). Skeletal morbidity in childhood acute lymphoblastic leukaemia. Clin Endocrinol.

[31.••] te Winkel ML, Pieters R, Hop WC (2014). Bone mineral density at diagnosis determine fracture rates in children with acute lymphoblastic leukemia treated according to the DCOG-ALL9 protocol. Bone.

[32] Ragab AH, Frech RS, Vietti TJ (1970). Osteoporotic fractures secondary to methotrexate therapy of acute leukemia in remission. Cancer.

[33] Davies JH, Evans BA, Jenney ME, Gregory JW (2003). Effects of chemotherapeutic agents on the function of primary human osteoblast-like cells derived from children. J Clin Endocrinol Metab.

[34] Kohler JA, Moon RJ, Sands R (2012). Selective reduction in trabecular volumetric bone mineral density during treatment for childhood acute lymphoblastic leukemia. Bone.

[35] Boot AM, van den Heuvel-Eibrink MM, Hahlen K (1999). Bone mineral density in children with acute lymphoblastic leukaemia. Eur J Cancer.

[36] Davies JH, Evans BA, Jones E (2004). Osteopenia, excess adiposity and hyperleptinaemia during 2 years of treatment for childhood acute lymphoblastic leukaemia without cranial irradiation. Clin Endocrinol.

[37.••] Mostoufi-Moab S, Brodsky J, Isaacoff EJ (2012). Longitudinal assessment of bone density and structure in childhood survivors of acute lymphoblastic leukemia without cranial radiation. J Clin Endocrinol Metab.

[38] van der Sluis IM, van den Heuvel-Eibrink MM, Hahlen K (2000). Bone mineral density, body composition, and height in long-term survivors of acute lymphoblastic leukemia in childhood. Med Pediatr Oncol.

[39] Atkinson SA, Fraher L, Gundberg CM (1989). Mineral homeostasis and bone mass in children treated for acute lymphoblastic leukemia. J Pediatr.

[40] Elmantaser ME, Young D, Gibson B, Ahmed SF (2011). Skeletal morbidity in children receiving chemotherapy for acute lymphoblastic leukemia and its association with mineral homeostasis and duration of inpatient stay. J Pediatr Hematol Oncol.

[41] Crofton PM, Ahmed SF, Wade JC (1998). Effects of intensive chemotherapy on bone and collagen turnover and the growth hormone axis in children with acute lymphoblastic leukemia. J Clin Endocrinol Metab.

[42] Atkinson SA, Halton JM, Bradley C (1998). Bone and mineral abnormalities in childhood acute lymphoblastic leukemia: influence of disease, drugs and nutrition. Int J Cancer Suppl.

[43] Fatemi S, Ryzen E, Flores J (1991). Effect of experimental human magnesium depletion on parathyroid hormone secretion and 1,25-dihydroxyvitamin D metabolism. J Clin Endocrinol Metab.

[44] Specker B, Binkley T (2003). Randomized trial of physical activity and calcium supplementation on bone mineral content in 3- to 5-year-old children. J Bone Miner Res.

[45] Fuchs RK, Bauer JJ, Snow CM (2001). Jumping improves hip and lumbar spine bone mass in prepubescent children: a randomized controlled trial. J Bone Miner Res.

[46] Kaste SC, Qi A, Smith K (2014). Calcium and cholecalciferol supplementation provides no added benefit to nutritional counseling to improve bone mineral density in survivors of childhood acute lymphoblastic leukemia (ALL). Pediatr Blood Cancer.

[47] Lethaby C, Wiernikowski J, Sala A (2007). Bisphosphonate therapy for reduced bone mineral density during treatment of acute lymphoblastic leukemia in childhood and adolescence: a report of preliminary experience. J Pediatr Hematol Oncol.

[48] Weinstein RS (2011). Clinical practice. Glucocorticoid-induced bone disease. N Engl J Med.

[49] Nysom K, Holm K, Michaelsen KF (1998). Bone mass after treatment for acute lymphoblastic leukemia in childhood. J Clin Oncol.

[50] Strauss AJ, Su JT, Dalton VM (2001). Bony morbidity in children treated for acute lymphoblastic leukemia. J Clin Oncol.

[51.••] Wilson CL, Dilley K, Ness KK (2012). Fractures among long-term survivors of childhood cancer: a report from the Childhood Cancer Survivor Study. Cancer.

[52] Marinovic D, Dorgeret S, Lescoeur B (2005). Improvement in bone mineral density and body composition in survivors of childhood acute lymphoblastic leukemia: a 1-year prospective study. Pediatrics.

[53] Kaste SC, Jones-Wallace D, Rose SR (2001). Bone mineral decrements in survivors of childhood acute lymphoblastic leukemia: frequency of occurrence and risk factors for their development. Leukemia.

[54] Thomas IH, Donohue JE, Ness KK (2008). Bone mineral density in young adult survivors of acute lymphoblastic leukemia. Cancer.

[55] Makitie O, Heikkinen R, Toiviainen-Salo S (2013). Long-term skeletal consequences of childhood acute lymphoblastic leukemia in adult males: a cohort study. Eur J Endocrinol.

[56] Gurney JG, Kaste SC, Liu W (2014). Bone mineral density among long-term survivors of childhood acute lymphoblastic leukemia: results from the St. Jude Lifetime Cohort Study. Pediatr Blood Cancer.

[57] Baek KH, Lee WY, Oh KW (2004). Changes in the serum growth factors and osteoprotegerin after bone marrow transplantation: impact on bone and mineral metabolism. J Clin Endocrinol Metab.

[58] Hartman A, van den Bos C, Stijnen T, Pieters R (2008). Decrease in peripheral muscle strength and ankle dorsiflexion as long-term side effects of treatment for childhood cancer. Pediatr Blood Cancer.

[59] Benker G, Schafer U, Hermanns U (1989). Allogenic bone marrow transplantation in adults: endocrine sequelae after 1-6 years. Acta Endocrinol (Copenh).

[60] Lee WY, Baek KH, Rhee EJ (2004). Impact of circulating bone-resorbing cytokines on the subsequent bone loss following bone marrow transplantation. Bone Marrow Transplant.

[61.•] Mostoufi-Moab S, Ginsberg JP, Bunin N (2012). Bone density and structure in long-term survivors of pediatric allogeneic hematopoietic stem cell transplantation. J Bone Miner Res.

[62] Savani BN, Donohue T, Kozanas E (2007). Increased risk of bone loss without fracture risk in long-term survivors after allogeneic stem cell transplantation. Biol Blood Marrow Transplant.

[63] Di Iorgi N, Muratori T, Secco A (2008). Quantitative ultrasound detects bone impairment after bone marrow transplantation in children and adolescents affected by hematological diseases. Bone.

[64] Bhatia S, Ramsay NK, Weisdorf D (1998). Bone mineral density in patients undergoing bone marrow transplantation for myeloid malignancies. Bone Marrow Transplant.

[65] Taskinen M, Kananen K, Valimaki M (2006). Risk factors for reduced areal bone mineral density in young adults with stem cell transplantation in childhood. Pediatr Transplant.

[66] Valimaki MJ, Kinnunen K, Volin L (1999). A prospective study of bone loss and turnover after allogeneic bone marrow transplantation: effect of calcium supplementation with or without calcitonin. Bone Marrow Transplant.

[67] Kaste SC, Shidler TJ, Tong X (2004). Bone mineral density and osteonecrosis in survivors of childhood allogeneic bone marrow transplantation. Bone Marrow Transplant.

[68] Nysom K, Holm K, Michaelsen KF (2000). Bone mass after allogeneic BMT for childhood leukaemia or lymphoma. Bone Marrow Transplant.

[69] Petryk A, Bergemann TL, Polga KM (2006). Prospective study of changes in bone mineral density and turnover in children after hematopoietic cell transplantation. J Clin Endocrinol Metab.

[70] Perkins JL, Kunin-Batson AS, Youngren NM (2007). Long-term follow-up of children who underwent hematopoietic cell transplant (HCT) for AML or ALL at less than 3 years of age. Pediatr Blood Cancer.

[71] Zemel BS, Leonard MB, Kelly A (2010). Height adjustment in assessing dual energy x-ray absorptiometry measurements of bone mass and density in children. J Clin Endocrinol Metab.

[72] Dubner S, Shults J, Baldassano RN (2009). Longitudinal assessment of trabecular and cortical bone density and structure in an incident cohort of children with Crohn’s disease. Gastroenterology.

[73] Burnham JM, Shults J, Dubner SE (2008). Bone density, structure, and strength in juvenile idiopathic arthritis: importance of disease severity and muscle deficits. Arthritis Rheum.

[74] Wetzsteon RJ, Kalkwarf HJ, Shults J, et al. Volumetric bone mineral density and bone structure in childhood chronic kidney disease. J Bone Miner Res. 2011;26(9):2235–44. 10.1002/jbmr.427 PMC3304436 21590737

[75] Burger B, Beier R, Zimmermann M (2005). Osteonecrosis: a treatment related toxicity in childhood acute lymphoblastic leukemia (ALL)—experiences from trial ALL-BFM 95. Pediatr Blood Cancer.

[76] Campbell S, Sun CL, Kurian S (2009). Predictors of avascular necrosis of bone in long-term survivors of hematopoietic cell transplantation. Cancer.

[77] Brown KR, Rzucidlo E (2011). Acute and chronic radiation injury. J Vasc Surg.

[78] Willems E, Baron F, Seidel L (2010). Comparison of thrombotic microangiopathy after allogeneic hematopoietic cell transplantation with high-dose or nonmyeloablative conditioning. Bone Marrow Transplant.

[79] Lafforgue P (2006). Pathophysiology and natural history of avascular necrosis of bone. Joint Bone Spine: revue du rhumatisme.

[80] Mitchell CD, Richards SM, Kinsey SE (2005). Benefit of dexamethasone compared with prednisolone for childhood acute lymphoblastic leukaemia: results of the UK Medical Research Council ALL97 randomized trial. Br J Haematol.

[81.••] Kawedia JD, Kaste SC, Pei D, et al. Pharmacokinetic, pharmacodynamic, and pharmacogenetic determinants of osteonecrosis in children with acute lymphoblastic leukemia. Blood. 2011;117(8):2340–7. 10.1182/blood-2010-10-311969 PMC3062406 21148812

[82] Mattano LA, Sather HN, Trigg ME, Nachman JB (2000). Osteonecrosis as a complication of treating acute lymphoblastic leukemia in children: a report from the Children’s Cancer Group. J Clin Oncol.

[83] Miettunen PM, Lafay-Cousin L, Guilcher GM (2012). Widespread osteonecrosis in children with leukemia revealed by whole-body MRI. Clin Orthop Relat Res.

[84] Padhye B, Dalla-Pozza L, Little DG, Munns CF (2013). Use of zoledronic acid for treatment of chemotherapy related osteonecrosis in children and adolescents: a retrospective analysis. Pediatr Blood Cancer.

[85] Karimova EJ, Rai SN, Howard SC (2007). Femoral head osteonecrosis in pediatric and young adult patients with leukemia or lymphoma. J Clin Oncol.

[86] Ojala AE, Paakko E, Lanning FP, Lanning M (1999). Osteonecrosis during the treatment of childhood acute lymphoblastic leukemia: a prospective MRI study. Med Pediatr Oncol.

[87.••] te Winkel ML, Pieters R, Hop WC (2011). Prospective study on incidence, risk factors, and long-term outcome of osteonecrosis in pediatric acute lymphoblastic leukemia. J Clin Oncol.

[88] Niebrugge DJ, Benjamin DR (1983). Bone marrow necrosis preceding acute lymphoblastic leukemia in childhood. Cancer.

[89] Arico M, Boccalatte MF, Silvestri D (2003). Osteonecrosis: an emerging complication of intensive chemotherapy for childhood acute lymphoblastic leukemia. Haematologica.

[90] Elmantaser M, Stewart G, Young D (2010). Skeletal morbidity in children receiving chemotherapy for acute lymphoblastic leukaemia. Arch Dis Child.

[91] Mattano LA, Devidas M, Nachman JB (2012). Effect of alternate-week vs continuous dexamethasone scheduling on the risk of osteonecrosis in paediatric patients with acute lymphoblastic leukaemia: results from the CCG-1961 randomized cohort trial. Lancet Oncol.

[92] Relling MV, Yang W, Das S (2004). Pharmacogenetic risk factors for osteonecrosis of the hip among children with leukemia. J Clin Oncol.

[93] Ribeiro RC, Fletcher BD, Kennedy W (2001). Magnetic resonance imaging detection of avascular necrosis of the bone in children receiving intensive prednisone therapy for acute lymphoblastic leukemia or non-Hodgkin lymphoma. Leukemia.

[94] Hyakuna N, Shimomura Y, Watanabe A (2014). Assessment of corticosteroid- induced osteonecrosis in children undergoing chemotherapy for acute lymphoblastic leukemia: a report from the Japanese Childhood Cancer and Leukemia Study Group. J Pediatr Hematol Oncol.

[95] Vrooman LM, Stevenson KE, Supko JG (2013). Postinduction dexamethasone and individualized dosing of Escherichia Coli L-asparaginase each improve outcome of children and adolescents with newly diagnosed acute lymphoblastic leukemia: results from a randomized study—Dana-Farber Cancer Institute ALL Consortium Protocol 00-01. J Clin Oncol.

[96] Vora A, Wade R, Mitchell C, et al. Incidence and outcome of osteonecrosis in children and young adults with acute lymphoblastic leukaemia treated on a dexamethasone containing protocol: results of the Medical Research Council UK trial ALL 2003. Blood. 2008;112:A910.

[97] Moricke A, Zimmerman M, Schrauder A, et al. No influence on the incidence of osteonecroses when dexamethasone replaces prednisone during induction treatment for childhood ALL: results of trial ALL-BFM 2000. Blood. 2008;112:899 [Abstract].

[98] Mattano LA Jr, Nachman JB, Devidas M, et al. Increased incidence of osteonecrosis (ON) with a dexamethasone (DEX) induction for high risk acute lymphoblastic leukemia (HR-ALL): a report from the Children's Oncology Group (COG). Blood. 2008;112:A898.

[99] Niinimaki RA, Harila-Saari AH, Jartti AE (2007). High body mass index increases the risk for osteonecrosis in children with acute lymphoblastic leukemia. J Clin Oncol.

[100] French D, Hamilton LH, Mattano LA (2008). A PAI-1 (SERPINE1) polymorphism predicts osteonecrosis in children with acute lymphoblastic leukemia: a report from the Children’s Oncology Group. Blood.

[101] Asano T, Takahashi KA, Fujioka M (2003). Genetic analysis of steroid-induced osteonecrosis of the femoral head. J Orthop Sci.

[102] Bond J, Adams S, Richards S (2011). Polymorphism in the PAI-1 (SERPINE1) gene and the risk of osteonecrosis in children with acute lymphoblastic leukemia. Blood.

[103] Kechli AM, Wilimas JA, Pui CH (1999). Factor V Leiden and other hypercoagulable state mutations are not associated with osteonecrosis during or after treatment for pediatric malignancy. J Pediatr.

[104] Enright H, Haake R, Weisdorf D (1990). Avascular necrosis of bone: a common serious complication of allogeneic bone marrow transplantation. Am J Med.

[105] Ojala AE, Lanning FP, Paakko E, Lanning BM (1997). Osteonecrosis in children treated for acute lymphoblastic leukemia: a magnetic resonance imaging study after treatment. Med Pediatr Oncol.

[106] Faraci M, Calevo MG, Lanino E (2006). Osteonecrosis after allogeneic stem cell transplantation in childhood. A case-control study in Italy. Haematologica.

[107] Socie G, Cahn JY, Carmelo J (1997). Avascular necrosis of bone after allogeneic bone marrow transplantation: analysis of risk factors for 4388 patients by the Societe Francaise de Greffe de Moelle (SFGM). Br J Haematol.

[108] Tauchmanova L, De Rosa G, Serio B (2003). Avascular necrosis in long-term survivors after allogeneic or autologous stem cell transplantation: a single center experience and a review. Cancer.

[109] Schulte CM, Beelen DW (2004). Avascular osteonecrosis after allogeneic hematopoietic stem-cell transplantation: diagnosis and gender matter. Transplantation.

[110] Israelite C, Nelson CL, Ziarani CF (2005). Bilateral core decompression for osteonecrosis of the femoral head. Clin Orthop Relat Res.

[111] Barr RD, Sala A (2008). Osteonecrosis in children and adolescents with cancer. Pediatr Blood Cancer.

[112] Yen CY, Tu YK, Ma CH (2006). Osteonecrosis of the femoral head: comparison of clinical results for vascularized iliac and fibula bone grafting. J Reconstr Microsurg.

[113] Lieberman JR, Conduah A, Urist MR (2004). Treatment of osteonecrosis of the femoral head with core decompression and human bone morphogenetic protein. Clin Orthop Relat Res.

[114] Wei SY, Esmail AN, Bunin N, Dormans JP (2000). Avascular necrosis in children with acute lymphoblastic leukemia. J Pediatr Orthop.

[115] Trancik T, Lunceford E, Strum D (1990). The effect of electrical stimulation on osteonecrosis of the femoral head. Clin Orthop Relat Res.

[116] Steinberg ME, Brighton CT, Bands RE, Hartman KM (1990). Capacitive coupling as an adjunctive treatment for avascular necrosis. Clin Orthop Relat Res.

[117] Bernbeck B, Christaras A, Krauth K (2004). Bone marrow oedema and aseptic osteonecrosis in children and adolescents with acute lymphoblastic leukaemia or non-Hodgkin-lymphoma treated with hyperbaric-oxygen-therapy (HBO): an approach to cure?—BME/AON and hyperbaric oxygen therapy as a treatment modality. Klin Padiatr.

[118] Jager M, Zilkens C, Westhoff B (2009). Efficiency of iloprost treatment for chemotherapy-associated osteonecrosis after childhood cancer. Anticancer Res.

[119] Li X, Cui Q, Kao C (2003). Lovastatin inhibits adipogenic and stimulates osteogenic differentiation by suppressing PPARgamma2 and increasing Cbfa1/Runx2 expression in bone marrow mesenchymal cell cultures. Bone.

[120] Pritchett JW (2001). Statin therapy decreases the risk of osteonecrosis in patients receiving steroids. Clin Orthop Relat Res.

[121] Laroche M, Jacquemier JM, Montane de la Roque P (1990). Nifedipine per os relieves the pain caused by osteonecrosis of the femur head. Revue du rhumatisme et des maladies osteo-articulaires.

[122] Aigner N, Petje G, Steinboeck G (2001). Treatment of bone-marrow oedema of the talus with the prostacyclin analogue iloprost. An MRI-controlled investigation of a new method. J Bone Joint Surg (Br).

[123] Agarwala S, Jain D, Joshi VR, Sule A (2005). Efficacy of alendronate, a bisphosphonate, in the treatment of AVN of the hip. A prospective open-label study. Rheumatology.

[124] Agarwala S, Shah S, Joshi VR (2009). The use of alendronate in the treatment of avascular necrosis of the femoral head: follow-up to eight years. J Bone Joint Surg (Br).

[125] Cardozo JB, Andrade DM, Santiago MB (2008). The use of bisphosphonate in the treatment of avascular necrosis: a systematic review. Clin Rheumatol.

[126] Ramachandran M, Ward K, Brown RR (2007). Intravenous bisphosphonate therapy for traumatic osteonecrosis of the femoral head in adolescents. J Bone Joint Surg Am.

[127] Rogers MJ (2003). New insights into the molecular mechanisms of action of bisphosphonates. Curr Pharm Design.

[128.•] Kotecha RS, Powers N, Lee SJ (2010). Use of bisphosphonates for the treatment of osteonecrosis as a complication of therapy for childhood acute lymphoblastic leukaemia (ALL). Pediatr Blood Cancer.

[129] Nguyen T, Zacharin MR (2006). Pamidronate treatment of steroid associated osteonecrosis in young patients treated for acute lymphoblastic leukaemia—two- year outcomes. J Pediatr Endocrinol Metab.

[130] Oeffinger KC, Mertens AC, Sklar CA (2006). Chronic health conditions in adult survivors of childhood cancer. N Engl J Med.

[131] Little DG, McDonald M, Sharpe IT (2005). Zoledronic acid improves femoral head sphericity in a rat model of Perthes disease. J Orthop Res.

